# Transformative Impact of the Internet on the Boundaries for the Physician Profession: Why Materiality Matters

**DOI:** 10.2196/63305

**Published:** 2025-09-22

**Authors:** Lena Petersson

**Affiliations:** 1 School of Health and Welfare, Halmstad University, Box 823, Halmstad, 301 18, Sweden, 46 702055024

**Keywords:** physicians, profession, jurisdiction, boundaries, professional knowledge, digital health technology, materiality, artificial intelligence, Open Notes, PatientsLikeMe, Apple watch

## Abstract

Over the last 25 years, the health care sector has undergone a digital transformation; health issues and medical conditions are increasingly managed with the support of digital health technology. The internet has transformed the boundaries around physicians’ work, which raises questions about how technological artifacts are transforming the boundaries that have traditionally existed between the health care professions and patients regarding information and knowledge. This viewpoint paper analyzes how digital health technologies can transform the boundaries of physicians’ work by examining 3 examples of technology aimed at patients or citizens: Open Notes, PatientsLikeMe, and Apple Watch. Traditionally, the physician profession drew the boundaries that separated it from other professions and patients to define and protect its jurisdiction and professional knowledge. However, in the 3 artifacts analyzed, technology changes the boundaries between laypeople and physicians. Therefore, health technologies aimed at citizens impact health care and its professions, and the materiality of artifacts can change the boundaries between physicians and citizens. Thus, the initiators and developers of technology aimed at patients or citizens may have the power to transform the field of knowledge in health care.

## Introduction

In many countries, digitalization in the health care sector leads to new requirements and opportunities for citizens, health care professionals, health care providers, and other societal actors. Today, health issues and medical conditions are often managed with support from digital technology. This raises the following questions: How does digital health technology transform how the information about, and knowledge of, citizens’ health and medical conditions are created? Where is the knowledge located? Who has access to it? What happens when we move from human to artificial intelligence (AI) to analyze health information? These questions are discussed in this viewpoint paper, based on 3 artifacts that shed light on how the materiality of digital technology impacts citizens’ access to information and knowledge about their health and that of others. This viewpoint paper clarifies how 3 digital health technologies—Open Notes, PatientsLikeMe (PLM), and Apple Watch—can transform the boundaries of work in the physician profession and, thus, the health care sector.

Health care has a long history of knowledge monopoly by the health care professions. Historically, it has been difficult for citizens to access information on health and medical conditions. Such knowledge was primarily found in medical books and scientific journals and could only be accessed through health care professionals who had undergone extensive academic education and training and had accumulated a wealth of practical experience [1,2]. Their tasks and responsibilities set them apart from laypeople, such as patients and citizens. Their roles and the accompanying expectations of their knowledge have been evident in health care for many years. There has always been an asymmetric relationship between physicians and patients, given physicians’ superior knowledge of health matters [3]. The internet, however, has transformed these conditions. This raises questions about how technological artifacts are transforming the boundaries that have traditionally existed between the health care professions and patients regarding information and knowledge [4]. This viewpoint paper highlights how changes in patients’ access to information impact physicians, often the health care professionals most affected by the ongoing digitalization in health care [5,6].

The relationship between “information” and “knowledge” can be described as follows: information becomes knowledge when a person has received it and understands its contents. To understand information, it must be put into context [7]. Information and knowledge can thus be described as the content, and learning is how individuals assimilate the content. This paper focuses on the contents of the learning process—information and knowledge—and how the internet and various digital health technologies offer patients or citizens access to them. Digitalization has transformed the conditions for laypeople’s knowledge development, and this viewpoint paper highlights its consequences.

## Health Information, the Internet, and AI

The internet is a powerful tool, and the range of technologies has grown in line with its development [4]. The description of its development, enabled by a complex and changeable technology, is often based on 2 generations of the internet: Web 1.0 and Web 2.0 [8]. The terms Medicine 1.0 and Medicine 2.0 are used to provide a general understanding of how these internet generations have transformed health care and, thus, the conditions for patients to find information and interact in health-related areas [8].

Launched in the 1990s, Web 1.0 made large amounts of information available. Communication was 1-way, and citizens could not post their own material. The term Medicine 1.0 describes the technology that enables patients to access health information that authorities and companies, for example, provide through the internet [8]. Web 2.0, the second internet generation, allows 2-way communication, participation in communication platforms, and user-generated content related to health and illness. The term Medicine 2.0 describes this development [8].

Eysenbach [9] suggests that the second internet generation resulted in a significant transformation, because it gave patients entirely new opportunities to gather information from multiple, independent sources. Eysenbach [9] calls this the process in which the physician, as an expert, supplies the patient with information “intermediation” (the Latin prefix inter- means between). As a consequence of Medicine 2.0, the physician’s mediating role changes, which Eysenbach [10] suggests has led to a decreased need for the physician as an intermediary in the information process. Thus, the technical development of Web 2.0 has facilitated a breakdown of the old roles, enabling patients to search for information beyond the established authorities [9]. Instead, other people or artifacts around the patient, such as apps or wearables, can provide guidance and support the patient’s independent gathering of knowledge. Eysenbach [9] refers to this process as “apomediation” (the Latin prefix apo- means separated from). Thus, the development of the internet is a central element in, and a driver for, the transformation of the health care sector. At the same time, AI may be the technology that will fundamentally change health care and alter future work in the physician profession.

AI has been hypothesized to address many challenges in health care [11], and the sector has been described as one of AI’s most promising application areas [12,13]. AI applications are argued to improve health care quality through improvements in diagnostics, monitoring, access, advanced decision making, and virtual consultations [14,15]. Digitized data in different formats are available in health care settings to create information-driven care [16]. However, there are concerns that the emphasis on AI and data-driven decisions will overshadow the empathy, trust, and personalized care traditionally provided by human clinicians [17] and that it is vital that the core values in health care are preserved when AI is implemented [18]. Other concerns relate to data privacy, algorithmic biases, and unequal access to AI-driven health care, issues that need to be addressed when implementing AI [19]. Despite these challenges, research has anticipated that AI and precision medicine have the potential to revolutionize health care by identifying patients with special care needs through insights derived from large, complex datasets [20,21]. This potentially marks the emergence of an evidence-based practice 2.0 [22]. AI in artifacts such as smartwatches has the potential to bring positive health-related outcomes for citizens [23]. They can be used for monitoring, nudging, and prediction in the health care sector and can thus be an important tool in value-based health care [24]. However, the integration of these wearables into health care is still at an early stage, and there are several issues, such as data quality and the availability of the internet, that need to be managed [23].

Consequently, more research is required to understand how digital health technologies and AI can transform the boundaries of work in health care. A critical factor in this transformation involves how the materiality of these technologies can transform the traditionally established boundaries around the knowledge of, respectively, physicians and patients, and others. Thus, the materiality of technologies is a variable that affects the relationships between and the role structures of various parties [25] and, therefore, the future organization of health care.

The next sections present the theoretical perspectives that are key to the analyses and discussions in the paper. From a sociology of professions perspective, physicians are a classic profession with boundaries surrounding their knowledge monopoly. This, along with the design of digital health technologies and AI, changes the boundaries around this classic profession. This paper thus focuses on the role of technology in the transformation of health care, which requires descriptions and analyses of digital artifacts. The point of departure is that the materiality of an artifact exists independently of the individuals who encounter it. Yet, the affordances (possible actions offered by technology) and constraints (limitations imposed by technology) are not independent of the individual’s interaction with it. Depending on their role, individuals are introduced to an artifact whose materiality is already preconfigured for them [26].

## Professional Work, Knowledge, and Boundaries

Professions are the carriers of the highest knowledge in their field [2,27]. A fundamental reason for the existence of professionals is that they can make better assessments than laypeople. If they cannot, laypeople would not need to consult them, unless (in the case of physicians) they required a prescription, and the concept of professional authority would break down [28]. This line of reasoning is important for this viewpoint paper because, of course, one of the key functions of health care is the provision of access to physicians’ knowledge about health and medical conditions. Professional authority is a form of power based on expert knowledge, where laypeople submit to the professional’s assessment and leave the decisions to them in situations of uncertainty [27]. The content and terminology of such specific professional knowledge are so arcane that laypeople must consult professionals to access it.

The metaphor of boundaries can be used to understand how professionals relate to one another and those around them, as a fundamental part of the growth and development of professions is the creation and maintenance of boundaries surrounding a field of knowledge. Boundaries constitute professions, and even established professions, such as physicians, must manage the boundary processes around the main spheres of professional action: jurisdictions, expertise, and networks [29]. According to Fournier, a professional field of knowledge is always dynamic, implying that the boundaries surrounding it are not static. However, it is essential for professions to maintain three primary types of boundaries: (1) the boundaries between professional groups, (2) the boundaries between the profession and the market, and (3) the boundaries between the profession and laypeople [30]. This viewpoint paper focuses on the boundaries between physicians and laypeople and how digitalization is transforming these boundaries. The basis of the authority of a professional, according to Fournier, is the existence of boundaries between themselves and the laypeople they meet professionally in matters related to their professional field of knowledge [30]. When knowledge becomes a product available outside health care, the boundaries between physicians and citizens may disappear. The aura of mystique that has historically surrounded professional fields of knowledge and made laypeople dependent on professionals for access to such knowledge begins to dissipate. This can change the relationship between professionals and laypeople [30].

According to Parsons, the division of roles in health care is hierarchical, and the past principle has been that the physician had the expert knowledge and, thus, the authority in the field [3]. The term “jurisdiction” describes a profession’s control over the boundaries of a given field of knowledge and the right to perform specific tasks within that field. A physician’s jurisdiction is divided into 3 tasks that require professional knowledge: diagnosis, inference, and treatment [31]. Drawing boundaries to exclude other groups has traditionally been part of a profession’s claim to jurisdiction in its field of knowledge and has governed how the health care sector and its professions have developed and transformed. Fournier states that changes in professional boundaries can lead to professions being “transformed,” with boundaries being moved rather than disappearing. When the professional field of knowledge becomes accessible to laypeople, the need for the professional and their knowledge may increase, as informed laypeople have more questions and an increased need for answers [30]. Lupton [4] states that there is now an expectation that citizens take responsibility for their health and that patients should be capable of making the right health-related choices with the support of digital health technology. However, the idea that laypeople should be proactive, well-informed participants in their own health care can give rise to technology-focused individualism [32], and both patients and physicians are at risk of losing control over health information [33]. This development may change the boundaries of the authority that Brante describes as a key characteristic of professions [27]. When the physician’s exclusive knowledge base is accessible to laypeople through various digital health technologies, the boundaries in the health care sector and the balance of power between the profession and the patient or citizen will change, and a shift of power will occur.

## The Materiality of Technology

In this viewpoint paper, Leonardi’s [34] theoretical framework is used to analyze the materiality of technology. A starting point for this theory is that technologies are designed distinctly, and their design governs what they can be used for [34]. It is thus essential to study the affordances and constraints of various digital health technologies and their consequences for both patients and health care professionals. For example, apps increasingly provide visual representations to measure various health behaviors; these can be dynamic, allowing users to follow changes over time. Consequently, the design of technology offers individuals an opportunity to learn and increase their knowledge [7]. An increasing amount of information is stored and organized in various external memory systems (EMS) [7]. An EMS is a mediating artifact in which people’s experiences and knowledge are stored as information. These artifacts, designed by people, are not passive objects in the social contexts in which they are used. Those who design them have the power to enable or disable future actions within the artifacts. Such artifacts actively contribute to transforming information, and their design offers users various affordances [7], often discussed in relation to the artifact’s materiality [35]. The materiality of digital technology can thus transform possibilities for individuals, resulting in altered or entirely new ways of performing actions [25]. Digital artifacts have material characteristics, even though they cannot be physically touched. The material agency of digital artifacts is found in the design and programming of the artifact [35]. Thus, digital artifacts can be described as practical examples of how decision makers’ or entrepreneurs’ visions and ideas are realized in technical solutions. The 3 artifacts analyzed in this paper are digital health technologies for patients and citizens. The user affordances and constraints are thus based on the underlying visions and ideas that affect what can be done with the artifact [35]. The materiality of individual digital health technologies is significant because it governs what a patient or citizen can do with the technology. This, in turn, can affect and transform the boundaries between physicians and patients. Lupton [4] states that we need more knowledge of how the design and affordances of digital health technologies govern what people can do with them. Increasing patient empowerment is the basis for many recently developed digital health technologies. The authorities’ vision is that empowerment can be achieved by giving patients increased command over their health and the information connected to it [36]. This aligns with the endeavor to create digitally engaged patients [4].

The 3 digital health technologies were chosen to illustrate the distinct ways materiality offers patients or citizens information and, in some instances, knowledge about their health. This may change the boundaries between physicians and laypeople. The Swedish version of Open Notes is a civic health technology that gives patients online access to their electronic health records, promoted and deployed by the Swedish authorities. PLM is a digital patient support platform initiated and developed by an American company. The third technology is the wearable Apple Watch, from another American company. These 3 were selected because they constitute diverse examples [37], rather than for any general representation of digital health technologies. Their value lies in the fact that they are distinct from one another and thus reveal the diverse effects the materiality of digital artifacts can have on physicians’ work and, by extension, the organization of health care. Each technology is analyzed using Leonardi’s 3 cumulative steps for analyzing the role of technical artifacts in organizations [38]: (1) the first step considers the materials that produce the technological artifact. In a digital artifact, the materials can be the agency that the software routines afford the user or the technical prerequisites of the artifact; (2) the second step accounts for the materiality of the artifact in ways that link the materials to the affordances they produce; and (3) the third step accounts for the processes whereby these affordances materialize into practices that shape action and interaction that constitute organizing.

Information about the 3 health technologies was gathered from their websites, as well as from previously published information and research. The analyses were compared to reveal similarities and differences in the materiality of the artifacts.

## Three Health Technologies

This section presents and analyzes each health technology, focusing on how materiality changes the boundaries around physicians’ work. A table then presents a comparison of the 3 artifacts.

### Open Notes

#### Overview

Civic technology is at the interface between governments and citizens and can blur boundaries between actors [39]. In Sweden, the term Invånartjänst describes health care technology promoted and deployed by the authorities and aimed at citizens (ie, civic health technologies). One such technology is Open Notes, a civic service that gives patients online access to their electronic health records.

The Open Notes service in Sweden was created by employees of the Uppsala County Council and launched in 2012. Patients in nonpsychiatric care were offered access to their health records and several other civic health technologies via the internet [36]. At the request of central authorities, other county councils and regions followed suit. In 2017, all Swedish citizens aged 18 years and older were given online access to certain parts of their electronic health records, apart from nonpsychiatric care. By 2020, through the national Open Notes service, all Swedish citizens aged 16 years and older had access to all their information documented in county-financed health care and dental care.

The information a patient can see and when they can see it varies among the different county councils. Each patient can decide whether they want to read unsigned notes, which are notes that the responsible health care professional has not yet approved but are available online. A signed note means that the information is correct and officially approved. In some situations, health care professionals can enter a note that they have determined the patient should not have access to without a confidentiality check, for example, when the information entails a risk for a patient or their next of kin in a critical stage of treatment. The professional then uses a particular information template in Open Notes to ensure that such information is securely entered into the health records without the patient being able to see it.

#### Boundaries Opened by the Artifact

Open Notes is a Medicine 1.0 solution, where health care providers make information from health records digitally available through what Eysenbach [10] calls “intermediation.” This means that patients depend on the physician to enter information into the health records so that it can be mediated through Open Notes. While Open Notes constitutes an information source for patients, it also serves as a work tool for professionals, and the idea is that the electronic health record can serve both as a work tool for the profession and as a source of information for the patient.

This civic health technology exemplifies how county councils are altering the professional space of action for health care professions. The materiality of Open Notes blurs the boundaries between patient and professional by creating a transparent practice, where large amounts of information related to the work of the physician profession become visible when read by the patient, even before the professional has “finished thinking.” For instance, service design allows patients to read unsigned notes and test results that the physician has not yet reviewed. In 2023, 72% of Swedish citizens had read notes or test results in their health records online [40]. It is still too early to determine whether Open Notes will result in patients who are better informed and, if so, whether they will seek more health care services, as described by Fournier [30], based on the idea that they have more questions about their health.

Open Notes creates a transparent care practice by turning the individual physician’s expert knowledge into text through the writing of notes, thus also making the information available to the patient in real time through the technical solution. Keeping health records is a central part of physicians’ work. Thus, electronic health records are what Säljö [7] calls an EMS. The materiality of Open Notes breaks through the traditional boundary between the professional’s assessment and the patient’s access to this knowledge, which means that some of the knowledge becomes less exclusive. Changing the communication boundaries of the profession can, according to Fournier [30], lead to a dissipation of the mystique surrounding a professional field of knowledge. Such a change can lead to a transformation in the relationship between professionals and laypeople. Because of the Open Notes service, a patient no longer needs to contact a health care provider to request the contents of their health records in paper form or to ask an individual physician about their assessment. Instead, in the words of Säljö [7], the artifact mediates the information whenever the patient wants, which means that the patient has the same decision support as the professionals. This can lead to a reduction in the authority and legitimacy of the physician profession, as described by Brante [27] and Lupton [4], because the patient has access to and can question the assessments made based on information in the health records [6].

### PLM

#### Overview

PLM Inc, an American company, was established in 2004. In March 2006, the online meeting place PatientsLikeMe (PLM) was launched for patients with chronic and terminal diseases, amyotrophic lateral sclerosis. The aim was to help patients and their next of kin connect with others in the same situation to exchange information. In the next stage of development, the site was opened to patients with diagnoses such as HIV, AIDS, multiple sclerosis, epilepsy, Parkinson disease, and various mental health disorders. Since 2011, the site has been open to all patients, regardless of their diagnosis, and they can become members of PLM free of charge.

Members can discuss their medical conditions on the PLM website, interpret and describe their symptoms and experiences, and publish their medication plans. The contents are user-generated and consist solely of the experiences shared by members; no health care professionals interact with patients on the site. It is a patient-supported platform designed so that patients with any diagnosis can independently get information about their medical condition and contact other patients with the same condition. What sets PLM apart from many other online meeting places, according to Goetz [27], is that members not only share their experiences in posts but also quantify their symptoms and treatments using the underlying software. This converts large amounts of data into statistics and visual representations of each member’s health status. PLM has made the results of this quantification available for comparisons, and analyses are generated to increase learning and knowledge among the members [41]. At the time of writing (May 2025), according to their website, over 850,000 members were registered worldwide. Over 2800 conditions were covered, and 70 communities are offered to members. By comparison, in February 2018, over 2900 conditions were covered, and more than 600,000 members were registered on the website [42].

#### Boundaries Closed by the Artifact

PLM is an example of social networking in the health care area made possible by the technical development of the internet. PLM Inc is a highly successful Medicine 2.0 company operating in a global market. The technical development of Web 2.0 has created the conditions for increased knowledge sharing between patients and has given them increased learning opportunities. Leonardi and Barley [25] state that technology can change the conditions for individuals and enable them to perform previously impossible actions. Säljö [7] states that visualized representations offer knowledge through the collation of abstract connections. The materiality of PLM lets patients perform analyses and comparisons without being dependent on health care professionals. The service enables the exchange of experiences and overviews by the quantification and visualization of a patient’s individual disease progress and that of others. This affordance of the technology can enable patients to develop lay expertise [43].

PLM Inc is a company that operates based on health data registered by its members. Van Dijck and Poell [44] state that there is a double-edged logic inherent to online health platforms such as PLM. While they afford personal solutions to medical problems, promising to contribute to the public good, they also serve as personalized data-driven services for the company’s customers. Fournier [30] states that the market logic in society can result in a dismantling of professionals’ fields of knowledge. Today, the market and its mechanisms govern much of the technical development of digital technologies such as PLM. PLM’s materiality enables patients to build up their own knowledge banks, similar to Säljö’s [7] EMSs. This can alter the boundaries between professionals and patients, since the former cannot access the patients’ knowledge banks. Thus, there could be information about the patient that is also of value to the physician, but the physician cannot access the information unless the patient chooses to share it. The site is also an example of the bottom-up process of apomediation, as described by Eysenbach [9]. In addition to the design of the artifact, which enables patients to assess large amounts of information, there are agents, in the form of other patients, who share information. One of the most essential affordances that the company provides its members is an online meeting place that facilitates the accumulation of large amounts of information that is, in turn, transformed into knowledge by the underlying software, as Säljö [7] describes.

Artifacts are not passive objects; their design actively impacts what users can do [7]. The design of the PLM site allows patients to independently gather information about their medical condition. With other patients, they can also build a knowledge base supported by the materiality of the site. Leonardi and Barley [25] state that a technology’s materiality can change conditions for people. The information the members register on the PLM site emanates from their own experiences as patients with a given diagnosis and from the information they have received from health care professionals regarding their treatments and medications. This has resulted in a knowledge shift from health care providers to PLM, through patient registrations on the site. Thus, PLM is an example of what Säljö [7] calls a social memory system, where human experience and knowledge are offloaded and externalized in an artifact accessible to others. Here, knowledge is structured, organized, and transformed into visualizations and collations of a patient’s health status. This information is only provided to the physician if the patient chooses.

According to Fournier [30], it is essential to the authority of a profession that it maintains boundaries around its own knowledge field. This reasoning presupposes that the profession can impact the boundaries, which is not the case in the PLM example. Instead, PLM’s materiality closes the boundary to the physician profession because the knowledge generated by the site’s software is not automatically accessible to the profession. Patients can instead build up their own exclusive knowledge base beyond the control of the physician profession. Patients can compare their treatment with that of other patients and, in some cases, conclude that their physician’s inference differs from that of the physicians of other patients with the same symptoms. This can result in questioning the authority and legitimacy of the profession. In the long term, this can alter the boundaries of physician jurisdiction, as described by Abbott [13], because PLM has a materiality that enables patients to make their own diagnoses and inferences. The only professional jurisdiction that remains would be the task of suggesting treatment and prescribing medications.

### Apple Watch

#### Overview

Apple Watch is a smartwatch designed by the American company Apple Inc. The first Apple Watch was released in April 2015, and, at the time of writing, the latest generation is the Apple Watch Series 10. The wearable has AI-powered apps, and the delivery of notifications to the user can support a healthy lifestyle and behavioral changes. The artifact can also be used as a tool provided to patients by health care professionals for patient monitoring and guidance [23]. The smartwatch has sensors that gather data; the information is analyzed and transformed into health features through the company’s algorithms. The sensors can passively track multiple aspects of health, such as heart rate monitoring, temperature, and fall detection. In addition, the user can actively track aspects of health by entering self-reported data, such as moods or menstrual cycles [24].

In the autumn of 2024, the company presented a new feature in the form of a breathing disturbances metric and sleep apnea notifications. According to the company, this development will add to the many ways the Apple Watch acts as an intelligent guardian for users’ health. Sleep apnea is a respiratory disorder characterized by repeated interruptions in breathing during sleep. When an individual wears the Apple Watch during sleep, it monitors breathing using the accelerometer. The artifact categorizes any disturbances as “elevated” or “not elevated.” If an individual consistently experiences “elevated” disturbances over 30 days, they will receive a notification indicating potential signs of sleep apnea. They will also receive information recommending that, if they have not already been diagnosed with sleep apnea, they consult a physician [45].

#### Boundaries Blurred by the Artifact

Apple Watch is based on AI and Eysenbach’s [9] concept of apomediation. Citizens are offered personalized health information, making Apple Watch an interesting artifact to examine because, based on its materiality, it can be seen as an example of the social concept that citizens are interested in taking responsibility for their own health [4,32]. Apple Watch can thus be described as a Medicine 3.0 technology. Medicine 3.0 focuses on proactive medicine that emphasizes prevention, personalization, the use of AI, and continuous monitoring [46].

The company’s vision is for citizens to use the Apple Watch to support a healthy lifestyle. Citizens can gather the information they want about themselves and create their own knowledge bank regarding health matters without the physician profession having access to that information unless the citizen chooses to share it. The design can change the boundaries between physicians and citizens because citizens may have more information than the health care professionals. Apple Watch is the only one of the three digital health technologies aimed at citizens interested in health and lifestyle matters. They will be offered measurement values related to lifestyle matters and diagnoses, such as sleep apnea, from the smartwatch using AI. This will give the citizen the ability to build their own knowledge base using measurement values generated by the apps, which is reminiscent of what PLM offers. The smartwatch will generate enormous amounts of information that is processed and transformed into knowledge by AI in the technology and stored in what Säljö [7] calls an EMS.

According to Fournier [30], it is rare for professional boundaries to disappear, and this does not occur with Apple Watch. Instead, the materiality of the wearable may create new kinds of fluid or blurred boundaries between the profession, the patient, and the technology. When, for instance, the algorithms can diagnose sleep apnea, there is a risk that there will be uncertainty about jurisdiction [28,31] and interference. This may mean that the legitimacy and authority of the professions will need to be constructed in a new way and that the relationship between professionals and laypeople will change when laypeople bring the output from the smartwatch to the meeting, as proposed by Eyal et al [33]. As Fournier [30] wrote, this relationship will change if the mystique and exclusivity surrounding professionals’ knowledge dissipates. This may well happen if an increasing amount of knowledge is generated by intelligent technology, such as the Apple Watch. This, in turn, can change the boundaries surrounding the authority of the physician profession, which, according to Brante [27], is a key characteristic of a profession.

## Digital Technology Changes Boundaries

This viewpoint paper has focused on illuminating how digital health technology can facilitate the capacity of patients and citizens to gather information themselves in ways that can lead to changes in the boundaries between them and the physician profession. Table 1 presents a comparative summary of how the materiality of the 3 artifacts changes the boundaries of the physician profession’s work in relation to patients and citizens. Rows 1‐5 in Table 1 relate to the materials from which the 3 artifacts were created. Row 6 relates to the materiality of the artifacts, and rows 7 and 8 describe the processes in health care where the affordances materialize in practice.

**Table 1.** Comparative summary of the materiality of the 3 digital health artifacts.

Question	Open Notes	PatientsLikeMe	Apple Watch
Who took the initiative to develop the artifact?	Civil servants at a public agency.	Owners of a private company.	Owners of a private company.
What are the apparent underlying visions?	Transparency and well-informed patients.	Democratization and self-management.	Self-management and personalized health.
What internet generation is the artifact based on?	Medicine 1.0	Medicine 2.0	Medicine 3.0
Who contributes information?	Health care provider (from patient data, tests, and examinations).	Patient and health care provider, through the information they have received from the patient, other patients with the same diagnosis, and artificial intelligence.	Citizen and artificial intelligence through the wearable.
Who has access to the information?	Health care providers and patients.	Patients, other patients with the same diagnosis, researchers, and the company.	Citizen and company.
What are the affordances to the patient or citizen?	Information and access to physician work tools with registered knowledge.	Processing, analyses, comparisons, and visualizations of information; ability to create a personal knowledge bank and a personalized health plan; technical, community, and psychological support.	Information and the ability to build a personal knowledge bank (personalized health) through artificial intelligence in the wearable.
How does the artifact transform the boundaries of the physician profession’s jurisdiction?	No Changes	Changes regarding diagnosis and inference.	Changes regarding diagnosis and inference.
How do the professional boundaries change?	The artifact opens boundaries.	The artifact closes boundaries.	The artifact blurs boundaries.

Drawing boundaries to distinguish professionals from other groups in society is a natural part of professional work [30]. What is apparent in the analyses presented here, however, are the ways in which the 3 digital artifacts draw boundaries in new ways. It is no longer the physician profession that sets the boundaries that distinguish physicians from patients or other professionals; instead, it is the artifacts that change the boundaries around the physician profession through the visions built into the materiality of each artifact. Visions of increased self-management and patient empowerment through democratization and transparency are programmed into the software and design of the 3 digital artifacts. Boundaries have always been important to professions [29,30]. They have been a way of dividing duties and responsibilities between different groups of employees in health care. The success of the boundary-creating efforts has given the physician profession control over specific fields of knowledge in health care, and physicians have maintained many of these boundaries for a long time [47]. However, in the 3 artifacts presented, technology draws the boundaries for the physician profession through the visions built into the materiality of each artifact. In other words, the organization that controls the materiality of an artifact has power over the behavior of the affected individuals (patients, citizens, and physicians). The result is a transfer of power over physicians’ work from the profession to the owner of the technology (public agencies and private companies).

In each of the 3 artifacts, the technology affords: (1) easy access to patients’ digital information from their health records, (2) the ability to create digital networks between patients with identical symptoms and the same diagnosis, and (3) personalized health information analyzed by AI. Leonardi [35,38] states that the materiality of digital artifacts consists of their underlying ideas and visions. These 3 artifacts reveal this, in that the materiality of technology provides information and facilitates transparency and self-management.

This new technology means that some of the knowledge that, previously, was exclusive to the physician profession is now accessible without the involvement of that profession, thanks to AI on patient support platforms such as PLM and wearables such as Apple Watch. It is important to note that, in these artifacts, it is not the physician profession that governs this development (ie, physicians do not set the boundaries vis-à-vis the patients). Thus, the artifacts’ materiality can create, move, and change the boundaries between the artifacts, the physicians, and the patients or citizens. This will change their role in health care, and the initiators and developers of new technology will have primary power over this transformation. This is an entirely new scenario. In 2 of the technologies, PLM and Apple Watch, it can result in a change in the knowledge jurisdiction of the physician profession. This blurs the boundaries and changes the traditional power balance between profession and patient, which, in the past, was based on the knowledge and information advantage of the professions [31]. However, professional identities and boundaries both shape and are shaped by new technology. Digitalization and implementation of various artifacts can change professional boundaries [48] and thus generate boundary work [29,49] among physicians. Relevant questions are: How and where is the expert knowledge in the health area produced, stored, and made manifest in our society? What will the physician profession’s role be in this development in the future? What kind of boundary work [29,49] will physicians conduct? The organization of the health care sector in society is based on its being a source of knowledge in a specific area. It offers a physical or digital place where it is possible to get in touch with professionals with the highest levels of knowledge relating to health and medicine. Thus, the argument in this viewpoint paper is that we are now seeing a renegotiation, and the 3 artifacts presented show that the conditions are changing regarding time and space.

The artifact produced by Swedish authorities (Open Notes) offers the patient access to the profession’s knowledge bank. In contrast, PLM offers the patient the ability to build their own knowledge bank together with other patients, and Apple Watch offers the citizen a personalized knowledge bank. An essential question for the future concerns ways to define the information or knowledge that patients produce and participate in as users of various digital health technologies. For example, how should we evaluate the knowledge in the measurement points about treatments and disease symptoms that the patients themselves have created on the digital platform or the output from the algorithms in the Apple Watch? Is it to be considered unverified health information or a new form of medical knowledge produced by lay experts [43] with the support of the materiality of the health services?

Thus, ongoing development in digital technology guides patients toward new behaviors [4,32], and this can affect professional authority in specific areas of knowledge, which is crucial for professions. For a long time, it has been a matter of course that health care organizations and their professionals own the information about a patient’s health and medical conditions and that these professionals create new knowledge in the sector. Digital health technologies can thus transform the relationship between physicians and patients, and physicians need education and training to be prepared for these changes [33]. The changes can have an impact on who produces knowledge, owns the data, has power over the knowledge, and who has the authority to set and move the boundaries in the health care sector.

## Conclusions

The analysis of the 3 artifacts has revealed a diffuse borderland in which the materiality of digital health technologies leads to flexible boundaries between the information and knowledge of professions, patients, and citizens. Initiators and developers of technology aimed at patients or citizens may have the power to transform the field of knowledge in health care. Fournier [30] states that the professional field of knowledge is constantly changing, resulting in shifts in the boundaries surrounding it. The summary in Table 1 shows that the three artifacts have a materiality that alters the boundaries surrounding professional knowledge, albeit in different ways. By analyzing these artifacts with the support of theories on materiality and professional boundaries, we can increase our understanding of how the development of digital health technologies changes the conditions in health care for the creation of boundaries, professional work, and the organization of health care.

## Abbreviations

AI: artificial intelligence
EMS: external memory system
PLM: PatientsLikeMe
